# Gender effects in allergology – Secondary publications and update

**DOI:** 10.1186/s40413-017-0178-8

**Published:** 2017-12-28

**Authors:** Erika Jensen-Jarolim

**Affiliations:** 1 Institute of Pathophysiology and Allergy Research, Center of Pathophysiology, Infectiology and Immunology, Medical University Vienna, Währinger G. 18-20, 1090 Vienna, Austria; 2 The Interuniversity Messerli Research Institute, University of Veterinary Medicine Vienna, Medical University Vienna, and University Vienna, Vienna, Austria; 3 AllergyCare, Allergy Diagnosis and Study Center Vienna, Vienna, Austria

## Editorial

There is manifold evidence that gender aspects are particularly interesting in allergology.

In this article collection you will find an update on selected topics, aiming to provide an overview of the latest state-of-the-art research. Since we last concentrated on this topic [1] it has “exploded”, prompting us to take it up again.

Men and women are different in their lifestyles, in regards to selection of a specific profession, in the type of sports performed, in the intake of hormonal medications, and finally in what they eat. The most significant effect, however, seems to be connected to the influence of sexual hormones themselves. Our immune cells express hormonal receptors and can thereby be greatly influenced by the body’s own, as well as by administered, sexual hormones.

*Koper, Hufnagl & Ehmann* focus on bronchial asthma in the context of gender medicine [2]. While at a young age more boys develop asthma, this changes rapidly with girls’ sexual maturation, leading to a lifelong female dominance in allergy. One gender aspect, for instance, is that additional obesity synergizes with asthma development in girls, while this is not the case in boys of the same age. Testosterone seems to exert a protective role, while estrogen aggravates asthma. This phenomenon accompanies women in waves throughout their lives, starting with the first menstrual cycle, continuing with the intake of birth control pills, pregnancy, and finally hormone replacement therapy. In addition, women are more receptive to environmental toxins such as smoking. Gender effects in asthma are documented in epidemiology, pathophysiology and symptom enhancement. As a result, alternative asthma medications are prescribed in female asthma patients.

In the second article, *Isabella Pali*-*Schöll* and *Sheriene M. Afifi* review the current evidence of gender effects in food allergy and food intolerance, which often is difficult to sort out for lay people [3]. In these cases – again in the context of estrogen – enhanced barrier permeability of female skin and mucosa could be decisive, in that more of a disease-eliciting food is taken up, leading to symptoms. The metabolic capacity of a woman is also lower. For exactly this reason it is so important to once again analyze the pathophysiological mechanisms, from IgE-mediated food allergy with a possible high risk of life-threatening reactions, to celiac disease, to histamine and lactose intolerance.

As is the case with asthma, more female patients are recorded with food allergies from sexual maturity on, pointing towards a direct negative effect of female sexual hormones. Additionally, more females develop more severe anaphylactic reactions, with a correlation to physical exercise. Interestingly, some allergens are more likely to elicit allergic reactions in females, such as berries and fruits. The latter clearly points towards a gender aspect in lifestyle affecting the pattern of clinical symptoms. To this end, the only possible intervention in food allergy, as well as in intolerances, is food avoidance. This is usually followed more stringently by women than men, but unfortunately without preceding exact diagnostics. This may drive the affected women into malnutrition.

Female sexual hormones elevate the risk and symptom levels in asthma and food allergies. Things become paradoxical if hypersensitivity is developed to one’s own hormones, in the sense of “hormone allergy”. This aspect is revealed in the overview article by *Eva Untersmayr* et al. [4].

It concerns a less appreciated topic, however, with a potentially great implication in clinical practice. Hormone substitution is indispensable in a modern woman’s life, in terms of family planning, in vitro fertilization, and anti-aging strategies. Hormone substitution is not just used to "remain young", but also to avoid decreasing hormonal levels which are an important contribution to osteoporosis. These un-physiologic, exogenous hormone supplies in particular seem to be the trigger for specific hypersensitivities, which are characterized by rather irregular symptoms: migraine, joint pain, eczema, and exacerbation of acne like dermatitis and dyspnea have been described.

Moreover, the immunologically mediated reaction is a cause for repeated miscarriages and pregnancy loss. These connections are seldom considered today, as there is no functional interaction and exchange established between allergists and gynecologists. This is a pity, as very specific diagnosis is possible and even specific desensitization protocols have been elaborated [5].

Similarly, in transgender medicine hormonal gavages for feminization (or virilization) play an important role. Notably, very high dosages are prescribed in this setting (e.g. sublingual 1–4 mg estradiol/day, or transdermal 100–200mcg estradiol/day, or -i.m. 10–20 mg estradiol i.m. every 1–2 weeks over a period of 2 years, as comparing to contraceptives with 0,03 mg/day only). While it is well known in the transgender scene that allergies and asthma are among the undesired side effects, there are today no controlled studies on this topic. In the “age of Conchita Wurst” we should face these facts and trends and be open to them.

*Monika Raulf and colleagues* dedicate their article to gender aspects in occupational allergies [6]. Here, the choice of the working place, which is essentially determined by environment and culture, immediately pre-disposes one for exposure to allergens. Gender-related topics include part-time jobs, two or more jobs, engagement in care of children and seniors, and “female professions, like in health care, textile industry, food production or haircutters”. These professions endanger the skin barrier and lead to an elevated risk of sensitizations to environmental and contact allergens, especially on the hands.

Therefore, we may conclude that the type of profession clearly determines the relevant allergen. For instance, women are more often affected by occupational skin diseases and they seek a doctor’s advice earlier than men. There is an interesting connection between obstructive lung diseases, among them exogenous allergic alveolitis, which typically is caused by other exogenous allergens in women that in men. Among elicitors for occupational hypersensitivities one also finds animal dander, as the animal keeper profession largely is in female hands, too. These occupational allergies often lead to sick days, and finally may prompt to exit strategies out of the profession. The loss of time due to necessary re-training for a new profession is an economic problem for the affected individual as well as for society.

The article collection on gender aspects in allergy is completed by an uncommon view: The perspective of our pets who are increasingly affected by allergies. From a knowledgeable viewpoint, one will realize with ease that four-legged family members may also suffer from atopic diseases, skin inflammation, unaesthetic hair loss, and excoriations by superinfections and scratching.

These allergic phenotypes are seen in our best friends: the dog, cat and horse. It may be expected that in the future new medicinal knowledge will be generated by comparative studies between “man and beast” (“comparative medicine”), facilitating the incovering of systematic health problems. In the present article collection *Ina Herrmann* et al. go one step further and analyze the evidence for gender aspects in allergies of domestic animals [7]. Their search was challenging as many of the animals are spayed or neutered, thereby diminishing all hormonal effects. Therefore in this young field, besides sporadic evidence, there is no proof yet whether more male or female pets develop allergies.

Could the allergens themselves be female or male? The answer is – yes! Some animal allergens are sex-dependent. The allergen Can f 5 of dog is a homologue of the human prostate-specific antigen (PSA) and is secreted via the urine. Therefore, patients sensitized to animal dander may react towards a male dog, but keep a female dog without any problems. The homology with human PSA can lead to clinically relevant cross-reactivities in the offended patient [8]. It remains to be answered whether this aspect still belongs to the topic of “gender medicine”.

Together, we draw a comprehensive thematic bow over gender aspects in allergy in this article collection, which hopefully represents an interesting overview for our (female and male) readers.

Erika Jensen-Jarolim, MD.

## References

[1] Jensen-Jarolim E, Untersmayr E (2008). Gender-medicine aspects in allergology. Allergy.

[2] Koper I, Hufnagl K, Ehmann R. Gender aspects and influence of hormones on bronchial asthma – Secondary publication and update. (Original: Die Einflüsse von Gender und Hormonen beim Asthma bronchiale. Allergologie. 2017;40(3):94-100). World Allergy Organ J. 2017;10:46. 10.1186/s40413-017-0177-9 PMC5745695 29308113

[3] Afify SM, Pali-Schöll I. Adverse reactions to food: the female dominance – A secondary publication and update. (Original: Nahrungsmittelunverträglichkeiten: die Dominanz der Frauen. Allergologie. 2017;40(3):101-10). World Allergy Organ J. 2017;10:43. 10.1186/s40413-017-0174-z PMC5746020 29308110

[4] Untersmayr E, Jensen AN, Walch K. Sex hormone allergy: clinical aspects, causes and therapeutic strategies – Update and secondary publication. (Original: Allergie gegen Hormone: Klinik, Ursachen und mögliche Behandlungsstrategien. Allergologie. 2017;40(3):111-6). World Allergy Organ J. 2017;10:45. 10.1186/s40413-017-0176-x PMC5745953 29308112

[5] Foer D, Buchheit KM, Gargiulo AR, Lynch DM, Castells M, Wickner PG (2016). Progestogen hypersensitivity in 24 cases: diagnosis, management, and proposed renaming and classification. J Allergy Clin Immunol Pract.

[6] Raulf M, Brüning T, Jensen-Jarolim E, Van Kampen V. Gender-related aspects in occupational allergies – Secondary publication and update. (Original: Berufliche Allergien: Inwieweit spielen Genderaspekte eine Rolle? Allergologie. 2017;40(3):117-2). World Allergy Organ J. 2017;10:44. 10.1186/s40413-017-0175-y PMC5745779 29308111

[7] Herrmann IP, Einhorn L, Panakova L. Gender aspects in allergies of pets – A secondary publication and update. (Original: Genderaspekte bei den Allergien unserer Haustiere. Allergologie. 2017;40(3):128-32). World Allergy Organ J. 2017;10:42. 10.1186/s40413-017-0172-1 PMC5746012 29308109

[8] Kofler L, Kofler H, Mattsson L, Lidholm J (2012). A case of dog-related human seminal plasma allergy. Eur Ann Allergy Clin Immunol.

